# Improved Methods for Fire Risk Assessment in Low-Income and Informal Settlements

**DOI:** 10.3390/ijerph14020139

**Published:** 2017-02-01

**Authors:** John Twigg, Nicola Christie, James Haworth, Emmanuel Osuteye, Artemis Skarlatidou

**Affiliations:** 1 Department of Civil, Environmental and Geomatic Engineering, University College London, London WC1E 6BT, UK; nicola.christie@ucl.ac.uk (N.C.); j.haworth@ucl.ac.uk (J.H.); a.skarlatidou@ucl.ac.uk (A.S.); 2 Development Planning Unit, University College London, London WC1E 6BT, UK; e.osuteye@ucl.ac.uk

**Keywords:** fire, risk assessment, geospatial technologies, low-income settlements, extensive risk

## Abstract

Fires cause over 300,000 deaths annually worldwide and leave millions more with permanent injuries: some 95% of these deaths are in low- and middle-income countries. Burn injury risk is strongly associated with low-income and informal (or slum) settlements, which are growing rapidly in an urbanising world. Fire policy and mitigation strategies in poorer countries are constrained by inadequate data on incidence, impacts, and causes, which is mainly due to a lack of capacity and resources for data collection, analysis, and modelling. As a first step towards overcoming such challenges, this project reviewed the literature on the subject to assess the potential of a range of methods and tools for identifying, assessing, and addressing fire risk in low-income and informal settlements; the process was supported by an expert workshop at University College London in May 2016. We suggest that community-based risk and vulnerability assessment methods, which are widely used in disaster risk reduction, could be adapted to urban fire risk assessment, and could be enhanced by advances in crowdsourcing and citizen science for geospatial data creation and collection. To assist urban planners, emergency managers, and community organisations who are working in resource-constrained settings to identify and assess relevant fire risk factors, we also suggest an improved analytical framework based on the Haddon Matrix.

## 1. Introduction

Fires cause over 300,000 deaths annually and are the fourth largest cause of accidental injury globally (after road accidents, falls, and drowning). Over 95% of the deaths and burn injuries are in low- and middle-income countries (LMICs), where death rates are nearly six times higher than in high-income countries [1,2]. The associated costs of damage to property and livelihoods in LMICs are likely to be considerable, but are often not recorded. Little is known about the incidence, impact, and causes of urban fires in these countries, particularly in lower-income and informal settlements, which are growing rapidly in an urbanising world. This is largely neglected as a policy issue, which is partly due to the lack of reliable data on incidence and impact at both national and local levels, coupled with inadequate financial, material, technical, and human capacities to act to reduce fire risk. Improved fire impact data and fire risk assessment tools are essential in enabling and stimulating decision makers to take action.

More than half of the world’s population now lives in urban centres. The majority of urban dwellers are in LMICs, where most future urban population growth is predicted to take place [3]. A high proportion of LMICs’ urban populations are in low-income and informal settlements: these by their nature are unplanned and often densely populated, with poor-quality housing, limited supporting infrastructure and services (including health care and emergency services), and high vulnerability to fires and other hazards [3]. Fires can start and spread easily in such locations due to a number of factors, including: cooking on open fires or unstable stoves, use of combustible fuels (for cooking, heating, and lighting), unsafe electrical connections, ignorance of safe practices, alcohol intoxication, arson, flammable building materials, overcrowding and high building density, lack of fire hydrants and water supplies, and the inability of fire services to bring fire-fighting equipment through narrow lanes and alleys. However, they are also the indirect product of broader socio-economic factors. Research over many years has demonstrated the association between residential fire incidence, the social and economic characteristics of residents, and housing and neighbourhood conditions [4,5]. Poverty and other forms of deprivation and marginalisation resulting from broader socio-economic trends, official policies, and planning decisions, generate conditions of vulnerability, contributing to poor housing quality, overcrowding, and failure to invest in protective measures [6,7].

Urban fires exemplify a global problem that the United Nations Office for Disaster Risk Reduction (UNISDR) defines as an “extensive risk”: i.e., the widespread risk “to repeated or persistent hazard conditions of low or moderate intensity, often of a highly localized nature, which can lead to debilitating cumulative disaster impacts” [8]. Data on the incidence and impact of urban fires in lower-income countries and communities are very limited and uneven, as we discuss below, and local authorities often have very little information on the built environment and populations in informal settlements [3]. However, there are many examples illustrating the nature and scale of the problem. Fires in January 2005, February 2008, and March 2009 in the Joe Slovo informal settlement in Cape Town, South Africa, destroyed over 3600 homes and made more than 13,000 people homeless [9]. In Old Fadama, the largest informal settlement in Accra, Ghana, with a population of about 80,000, a fire in May 2012 destroyed or damaged the homes of around 3500 people [10]. A fire in Valparaiso, Chile, in April 2014 destroyed some 2500 homes, with 12,500 people forced to evacuate: much of the destruction was in poorer and informal settlements in hillside ravines [7]. In Nepal, fires destroyed 38,924 homes between 1990 and 1996 [11].

Data from 2006 and 2009–2015 from DesInventar (an international disaster database discussed below) on disasters in the Western Area of Sierra Leone, the country’s most densely populated region and home to the capital Freetown, highlight fire outbreaks as the most prevalent of all disasters. Although road and maritime accidents accounted for the most deaths (41% of all deaths recorded), fire accounted for 65% of houses destroyed or damaged [12]. The full extent of damage to livelihoods, health, and quality of life from this destruction of houses, property, and public infrastructure is undocumented and can only be inferred. Between 2011 and 2015 there were 547 fire outbreaks in the Western Area, with residential fires comprising 87% of the total [13].

## 2. Fire Data Limitations and Challenges

Reliable data on fire incidence, impacts, and causal factors in poor and informal settlements are essential for designing appropriate intervention strategies. Such data are rarely available in LMICs. The Geneva Association’s World Fire Statistics Centre publishes statistics generated by national governments on fire losses, but this covers only a relatively small number of high-income countries and the information is collected principally to inform the insurance industry [14]. In contrast, wildfire data are collected in a number of countries. Wildfires are estimated to affect 3–4 million km^2^ of the global land surface each year: they can be major events with considerable economic impacts, but often have little direct impact on human settlements (human casualties are relatively low), and wildfire-urban interfaces tend to be in wealthier suburban districts. Nevertheless, they do receive the attention of policy makers, and there is a Global Wildfire Monitoring Centre at Freiburg University, established in 1998, that supports mitigation and preparedness initiatives by the UN and other international organisations [15,16].

Whitby (2015) reviewed a range of fire, health, disaster, and human settlements databases and datasets providing information at global, national, and sub-national levels, taking into consideration the quality of the data (event, situational, victim, economic) and data accessibility. She found a lack of data on fire hazards, impacts, and vulnerabilities, together with inconsistent and incompatible data collection frameworks for compilation of relevant fire data in LMICs [17]. One of the publicly accessible disaster databases reviewed by Whitby, DesInventar, can provide a general idea of the distribution of disasters, including ‘extensive’ events such as fire outbreaks, in certain LMICs, with some degree of subnational disaggregation at the regional, provincial, or district level [12]. However, it is still not possible to make accurate conclusions about specific locations of interest which are prone to fires, such as informal settlements or urban centres, due to insufficiently detailed or consistent data [18]. Moreover, most databases describe disaster losses without exploring their underlying drivers. This requires collecting different strands of data on social factors (e.g., age, gender, income, ability, migrant status), environmental factors (e.g., access to good-quality housing and basic services) and political and institutional factors related to planning and decision-making processes at different levels [19].

Where comprehensive data are lacking, more specific studies at city or lower levels have been carried out using a mix of research methods to assess the scale of the fire problem, identify key issues, and recommend improvements [20,21]. An exemplar of thorough and extensive data collection to support decision making and strategic planning is the Monitoring, Mapping and Analysis of Disaster Incidents in Southern Africa (MANDISA) project, which collected data on fires in Cape Town from 1990–2004. During this period 8787 fires affected 41,301 dwellings in the city’s rapidly growing informal settlements, with informal dwellings accounting for over half of all fires by 2005. Analysis of the MANDISA database demonstrated a significant increase in the number of incidents over the reporting period and identified a range of physical, climatological, socio-economic, and political factors contributing to fire incidence and impact [22,23,24].

Epidemiological studies based on data from local hospital admissions are the main and most reliable sources of data on the nature of burn injuries and injury trends [25,26,27], although comparison between studies can be complicated by their inconsistent use of terminology [28]. Burn injuries are only one consequence of fires, and burn injury datasets do not record other forms of loss or damage to property and livelihoods. Nonetheless, they provide insights into the locations of fires and the immediate causes of injury (e.g., cooking fires, upset kerosene lamps, electrical faults, other forms of accident, or arson). Indirect or underlying causal factors are less easily identified, although such studies can sometimes identify socio-economic characteristics of injured people (e.g., age, gender, literacy levels, economic status) and differences in housing type.

Wider inferences can be drawn from aggregated data on a larger scale, where these are available. Sanghavi et al., 2009 used medically certified causes of death and verbal autopsy surveys to generate a retrospective analysis of fire-related deaths nationally in India in 2001, finding that a significant proportion of such deaths were of young women between 16 and 34 years of age [29]. Stylianou et al., 2015 analysed data from England and Wales from 2003–2011, identifying a decline in burn mortality overall and a higher proportion of males than females receiving burn injuries [30]. Kazerooni et al., 2016 carried out a systematic review of academic and non-academic literature on fires in camps and settlements for refugees and displaced people worldwide, which found a 25-fold increase in the rate of settlement fires between 1990 and 2015 [31].

In recent years, statistical and geospatial analysis and modelling of fires have advanced considerably, due to more widespread application of geographical information systems (GIS), improvements in statistical software, and greater computing power [5,32]. A variety of such methods is reported in the literature [33,34,35,36,37,38,39]. However, until now such technologies have been applied mostly in higher-income countries, which have the resources and technical capacities to utilise them, and where relevant datasets (such as census and housing) are extensive, reliable, and publicly available. Useful work can be done in LMICs where sufficient evidence is available, particularly at the sub-national level. For example, Sufianto and Green, 2012 analysed data on 4200 fire incidents in the cities of Jakarta and Surabaya in Indonesia from 2002–2008, producing information and understanding relating to damage to buildings and property, immediate causes, casualties, fire service response times, and the timing of incidents and their correlation with weather conditions [40]. Maniruzzaman and Haque’s study, 2013 of the area served by the Mohammadpur fire station in Dhaka, Bangladesh, collected and analysed data over a three-year period concerning the locations of fires by land use category, immediate causes, duration, and fire service performance in attending and extinguishing fires [41].

In general, however, attempts to collect and analyse data in LMICs have encountered a range of problems relating to the incompleteness, poor quality, and inconsistency of records (often generated as incident reports by over-stretched fire crews), inadequate information management and storage skills and facilities, and limited human and financial resources. Informal settlements present particular challenges to accurate geo-referencing, due to factors such as the lack of formal street addresses, repeated changes to neighbourhood boundaries, structural alterations, and the addition of new informal buildings [22,23,24].

## 3. Alternative and Innovative Approaches to Fire Risk Assessment

In these conditions, where formal datasets and data-gathering capacities remain limited, researchers and disaster risk management practitioners have to find alternative and more pragmatic methods of evaluating fire risks. In this section we identify some promising, practical approaches to acquiring data for fire risk assessment in low-income and informal settlements in LMICs. For example, the Asian Urban Development Program applied specified observable criteria for a series of fire risk factors in the city of Vientiane, Lao People’s Democratic Republic: these included building material type, building density, fire sources and history, road and water accessibility, and fire services capacity. Through a simple ranking system these assessments were used to generate fire risk maps and zonation [42,43].

Community-based, participatory risk and vulnerability assessments have been widely used in disaster risk management programming worldwide in recent years. Although these assessments take many different approaches and forms, they all seek to identify and assess the hazards and risks that people face in their locality, their vulnerability and resilience to those risks, and their capacity to manage them. They should also take a holistic view, considering the range of environmental, economic, social, and other factors that generate risk. This forms a basis for action planning and interventions. A wide variety of information sources and information-gathering tools are used, both quantitative and qualitative, including secondary data collection, geospatial data (ranging from satellite images to transect walks and community mapping), environmental checklists, group and individual interviews, oral histories and timelines, seasonal calendars, wealth and preference ranking, scenarios and simulations [44]. Mixing quantitative and qualitative data is valuable, since some risk factors—the role of conflict and power struggles, for example [45]—are not easily captured by quantitative methods.

Community-based risk assessment is well suited to environments where formal data may be unavailable, difficult to collect, inconsistent, or of poor quality, and it is intended to stimulate local debate, priority setting, and action. It also draws heavily on local people’s knowledge and experience of their environment and society, which is acknowledged to be an important resource in community-based risk management [46,47]. Lambert and Allen highlight the potential of using Participatory Action Research (PAR) methodology to map out risks in informal settlements, which promotes what they refer to as the “plurality of knowledges” and a better understanding of the risk profile. The use of participatory mapping confronts the conventional way of mapping risk by public institutions in LMICs, which often overlooks the potential of knowledge co-production in the identification of small-scale hazards [48]. Participatory enumerations, which can be carried out on a large scale, provide a wealth of socio-economic, demographic, and built environment data, thereby strengthening informal settlement communities’ bargaining power when negotiating with formal authorities for improvements to housing and infrastructure [9].

Community risk and vulnerability assessments are typically used to identify and understand the broad spectrum of hazard threats facing communities before agreeing on more targeted interventions, but the method can be applied effectively to single hazards such as fires. For example, a project investigating fires in Cape Town’s Imizamo Yethu informal settlement held semi-structured interviews and focus group discussions with a range of community members and local stakeholders, exploring the following topics: causes, locations, frequency, and severity of fires; areas and people most at risk (with a particular focus on risks to children); warnings and public education; coping strategies and fire-fighting capacities; and knowledge and understandings of fire risk (immediate and underlying causes) and ways of reducing it. This qualitative data was backed up with census and aerial photography evidence to provide quantitative information on population, housing, services, and infrastructure, and analysis of the MANDISA database for fire history and trends [49,50]. A community risk assessment in Makola Market, Accra, Ghana, one of the country’s two biggest markets, where fire is a common problem, used a mix of methods including visual surveys, interviews, photographs, Global Positioning System (GPS) mapping, and fire service data review to produce an analysis of incidents, experiences of fires, causal factors, and viable local solutions to the problem. This project also hired and trained market traders as field researchers [51].

Where time and resources are limited, more rapid assessments can be undertaken. Recent risk assessments by fire and rescue specialists in long-term refugee camps in Dadaab, Kenya, and Ban Mae Surin, Thailand, included interviews, focus group discussions, and participatory hazard mapping; however, because of time constraints linked to security concerns, the assessments relied heavily on direct observation of the local geography, the camps’ building design and layouts, cooking and lighting practices, and provision of fire-fighting equipment [52].

Opportunities are opening up for communities to use emerging forms of Geographic Information (GI) collection and dissemination to assess fire risk. Goodchild, 2007 coined the term Volunteered Geographic Information (VGI) to describe the phenomenon of the growing popularity of GI, which has seen increasing numbers of volunteers collecting, creating, and disseminating it in various forms [53]. Haklay, 2013 observes that VGI activities range from “fun activities of locating summer holiday photographs to focused surveying in the aftermath of an earthquake” [54]. Importantly, the majority of these volunteers do not have or require the qualifications and skills of those who have traditionally been responsible for the creation of GI, such as cartographers or experts working for national mapping agencies. VGI activities are enabled not only due to technological developments (e.g., the Web and its current utilisation, mobile devices and smartphones, and GPS receivers), but also due to other social phenomena, such as the rise of education levels, and people’s growing interest in voluntarism and issues where collective action is required (e.g., environmental monitoring).

The significance that GI plays in various everyday contexts has resulted in the creation of hundreds of VGI tools and activities to support all sorts of spatial decision making, participatory planning, and what is often called “citizen science” (i.e., the participation of non-professional scientists in scientific research, usually by collecting field data) [54]. See et al., 2016 reviewed over a hundred applications of VGI in diverse contexts including ecology and citizen science, environmental monitoring, travel websites and the sharing of geo-referenced photographs, transport, and weather reporting [55]. VGI has also proved particularly valuable in the context of disaster management, to enable prevention, preparation, response, and recovery [56,57]. For example, crisis mapping (using data collected by people using sensors such as the GPS receivers built into their mobile devices) has been repeatedly used to demonstrate the societal benefits of crowdsourced information. Applications such as Ushahidi and the humanitarian OpenStreetMap are noteworthy in this category: Ushahidi is a non-profit company based in Nairobi, Kenya, which has created interactive information sites for a number of disasters, including the Kenyan election crisis of 2007–2008 and the Haiti and Chile earthquakes in 2010 [58,59]; OpenStreetMap is a UK-based collective project to create a free and open map of the world, which allows data such as hazards, roads, and buildings to be entered by volunteers using a range of data sources and information-gathering techniques [60,61]. During Hurricane Sandy in 2012, 6717 volunteers analysed almost 35,000 photographs in 48 h [62]. Bonney et al., 2014 argue that many existing citizen science projects may easily be expanded to provide the protocols and infrastructure (technological and in terms of volunteers’ networks) to enable scientifically sound data collection during and after disaster situations [63].

VGI in the context of disaster management is a relatively new area of activity and research, and there is a limited number of working examples available for review. Tools and applications in the broader context of disaster management tend to appear in post-disaster situations and disappear subsequently because contributors are recruited on the ground and mapping takes place internal to organizations [56]. Nevertheless, there are many successful examples of applications created in response to earthquakes, such as Haiti in 2010; floods, as in Queensland in 2011; and wildfires, the most notable example being the Santa Barbara wildfires of 2007–2009 where different VGI forms and tools were used in response to fire incidents. Several lessons were drawn on their effectiveness and implications [62,64,65].

Most studies in the wider context of VGI for disaster management study post-disaster incidents and there is limited evidence and research in the context of prevention and preparedness [62]. There is also limited evidence of how VGI can be effectively used in the context of fire risk assessment in urban informal settlements, where there are different technological implications and a lack of understanding of what data are useful. Yet several technologies exist, or could be easily extended or adapted, to support the collection and dissemination of GI to support the fire risk assessment process. For example, the Red Cross Earthquake app (developed by the American Red Cross, Washington, DC, USA) notifies its users when an earthquake occurs and provides them with an option to share their location and safety status. The QuakeFeed app (developed by Artisan Global LLC, Los Angeles, CA, USA) is another mobile-based application that uses GPS receivers to detect the location of a user and sends notifications about nearby earthquake incidents. Survey123 (developed by Esri, Redlands, CA, USA) and Epicollect+ (developed by Imperial College London, London, UK) resemble survey forms that allow users to contribute data with several qualitative characteristics and associated images. They can be modified to support any type of VGI activity. The Life360 app (developed by Life360, San Francisco, CA, USA) shows the location of a user and their family members and allows exchanging of messages, which can be particularly helpful in pre- and post-disaster situations.

VGI therefore has considerable potential for application to fire risk assessment in urban low-income or informal settlements. Important data that can be collected easily by the settlement’s community members and which can support prevention and preparedness include: the location of high risk areas; the location of activities that pose a high risk (as well as additional information with respect to these activities to understand when, why, and how frequently these occur); the identification and exact location of specific fire corridors within the settlement; and the location of water supplies. Consulting community members and groups, through participatory research and action planning, can support the identification of risk factors and subsequent data collection to reduce the probability of fire incidents (e.g., a community market trader association can collect data about market areas with high fire risk and locations of the relevant water supplies in these parts of the settlement).

Although existing applications and solutions could potentially support the collection of such datasets in urban low-income or informal settlements, barriers to application must be considered. These barriers include what is commonly referred to as the “digital divide” (i.e., inequalities in access to information and communications technologies due to social and economic status) and the lack of user skills and knowledge to use relevant technology, especially in conditions of poverty and challenging physical environments. Further research is needed in order to assess how effective VGI might be in these contexts and to understand how best to engage residents of informal settlements in VGI-based fire risk assessment and reduction initiatives. Nevertheless, there are already examples which demonstrate the successful development and use of VGI in other difficult environments, for instance, in the African rainforest [66].

Modern technology has also been used to enhance more traditional participatory mapping processes with local community actors in order to produce detailed risk profiles of communities. The traditional approach involves participants undertaking transect walks in the community to identify factors of interest (such as landmarks, hazards, safety mechanisms, and sites of previous disasters), plotting them onto a printed map and annotating the information being gathered. In addition to these observations, the stories and experiences of local dwellers are also documented. This process can be enhanced by training participants to use digital processes in a number of open-source mobile phone applications such as Epicollect+, MyTracks (developed by Google Inc., Mountain View, CA, USA), and Ramblr (developed by Imperial College London, UK), which helps with the parallel, systematic, and speedy collection of georeferenced data, whilst embedding pictorial, video, and audio files. Training provided for participants prepares them to use these tools and visualise the information gathered [48]. The use of the Ramblr mobile phone application as a parallel tool for data collection has been trialled in two informal settlements in Freetown, Sierra Leone to map risks, including fires, which were found to be prevalent in hillside settlements [67]. Data can be uploaded into publicly accessible online maps to share some of the non-confidential qualitative and quantitative aspects of the information gathered about risks in a particular locality [48].

Finally, it is noteworthy that few studies view low-income settlement fires within a broader analytical framework of risk, vulnerability, and resilience. This may act as a hindrance to more effective planning of data collection and interpretation of results, irrespective of data quantity and quality. Such conceptualisations are commonly applied to good effect to risk and vulnerability assessments carried out for disaster risk management programming more generally [68]. It is also surprising that more use is not made of the systematic approach developed by the US researcher William Haddon in the 1970s, originally for road accidents, which has subsequently been widely applied to understanding hazards and injuries in other contexts, including burn injuries [69,70,71,72]. The Haddon Matrix provides a framework for understanding the causes of injuries and helps identify ways to prevent them or limit the impact they have. The matrix looks at, and links, risk factors pre, during, and post event related to the person, the agent of harm, and the physical and social environment that can be addressed to prevent or minimise the impacts of a hazard. The World Health Organisation has applied the Haddon Matrix to burn injuries [2]. However, whilst this is a useful start, there are still a number of questions of how to apply it in low-income and informal settings. We have therefore amended the matrix as it has been applied to understanding burn injuries in formal settings, by including a set of questions (in italics) showing what needs to be asked to fill gaps in our understanding of fire prevention in informal and low-income settlements. These questions are derived from our review of the literature and the workshop discussions (see Table 1).

This exercise shows that there are many questions that need to be answered in order to populate the cells of a Haddon Matrix for the systemic analysis of fires and burn injuries in informal settlements. However, this process is a necessary prerequisite in order to develop risk mitigation strategies. Given the many unknowns, such strategies need to be developed using “bottom up” approaches such as community-based assessment as discussed above, or social marketing, to understand the barriers and motivators of fire risk prevention amongst the people facing such risks [73]. Such approaches can help residents generate the solutions themselves, working with facilitators to ensure that the strategies are achievable in the context of their everyday lives.

## 4. Conclusions

Fires are a frequent and significant “extensive” risk in urban environments, especially in unplanned and densely populated low-income or informal settlements, which are characterised by poor-quality housing and limited supporting infrastructure and services. Despite this, urban fires are neglected in disaster management policy and practice. One of the main reasons for this neglect is the lack of accurate, consistent, and comprehensive data on urban fire incidence and impact, both at national and local levels. Better data are essential to provide accurate risk assessments and inform effective planning to prevent, mitigate, and respond to fires. Improved evidence also helps to make the urban fire problem more prominent as a policy issue for decision makers, and to support advocacy on this issue by vulnerable communities.

It is difficult for countries and municipalities with limited financial, material, technical, and human capacities to collect and analyse evidence on urban fires. Formal epidemiological studies and statistical and geospatial analysis and modelling on this subject have been largely restricted to higher-income countries. Nevertheless, from our review of examples in both academic and operational literature, our study identifies a range of practical methods that can be used to collect and interpret (or reinterpret) information on fire risk in low-income and informal settlements. These include: developing simple observable criteria for fire risk factors; applying participatory risk assessment approaches (particularly participatory mapping), which are widely used in disaster risk management programming, to fire risk and vulnerability; and rapid visual assessments by technical specialists.

The widespread and rapidly increasing adoption of technologies for recording and sharing geospatial information appears to be particularly promising. Large numbers of volunteers around the world are collecting, creating, and disseminating such information in different forms to support spatial decision making, participatory planning, and “citizen science” in both development and crisis contexts. This phenomenon, known as VGI, is facilitated by numerous technological innovations including social media, the worldwide web, and smartphones. VGI appears to have considerable potential for application to community-based fire risk assessment in urban low-income or informal settlements. Technologies and practices exist, or could be adapted, to support this. However, further research and testing are needed to understand how effective VGI might be in these contexts and how best to engage residents. Barriers to adoption, such as inequality of access to new information and communications technologies, must also be considered.

Finally, we argue the need for more robust conceptual and analytical frameworks relating to fire risk, vulnerability, and resilience. This is essential for effective planning of data collection and for interpretation of the results. Such frameworks are commonly used in disaster risk management, but are not applied in the context of fires in low-income and informal urban settlements. We use the Haddon Matrix, originally developed for road accidents but subsequently adapted for burn injury, as an example of a framework that might be used in this context, but many other frameworks and conceptualisations could be considered.

## Figures and Tables

**Table 1:** Haddon Matrix (as applied to burn injury prevention by the World Health Organisation) adapted to show where information is needed for low-income/informal settlements.

	**Host**	**Agent/Vehicle**	**Physical Environment**	**Social Environment**
	(Children, Elderly, Adults in Home)	(Cigarette, Matches, Appliances, Heaters, and Upholstered Furniture)	(Home)	(Community Norms, Policies, Rules)
	Others such as those cooking in market stalls?	What are the key agents in informal settlements?	Where are the loci of fires in informal settlements?	What are the social structures in informal settlements and what are the implications of this for fire prevention?
Pre-event (before fire starts)	Teach children not to play with matches Provide information about fire risk and cooking (loose clothing, long hair, etc., may catch on fire) What sort of opportunities are there for behavioural change interventions in informal settlements?	Redesign cigarettes so they self-extinguish Automatic shut-off for appliances such as coffee makers. Inspect and clean chimneys, heating systems each year Would regular inspection work to address fire risks in an informal settlement? Who would do it?	Lower flammability of structures Insure adequate emergency escape exits from home How can the flammability of building materials be reduced in informal settlements? How can emergency exits be created in spatially constrained informal settlements?	Improve efforts to curb smoking initiation Improve smoking cessation efforts How can we improve the safety of the way people cook and store fuels to avoid fires in informal settlements?
Event (during fire)	Teach children to stop, drop, and roll Plan and practice a fire escape route with children and adults Teach children not to hide during a fire How can escape strategies be planned in informal settlements?	Design furniture with materials that are less toxic when burned Design upholstery that is flame resistant How can we ensure that materials used in informal settlements are flame resistant?	Install smoke detectors Install sprinklers Increase number of usable exits What warning devices are possible in informal settlements?	Pass ordinances requiring smoke detectors and/or sprinkler systems Fund the fire department adequately to provide enough personnel and equipment for rapid response Who has the responsibility as emergency responders in informal settlements?
Post-event (after child or person injured by fire)	Provide first aid and cardiopulmonary resuscitation (CPR) to all family members Who would provide first aid training in informal settings?	Design heaters with quick and easy shutoff device Who would provide replacement heaters/cooking appliances in informal settlements?	Build homes with less toxic building materials To what extent do informal settlements have toxic building materials and what alternatives are available?	Increase availability of burn treatment facilities What role do non-governmental organisations /charities play in providing such facilities in informal settlements?
